# Supplementary material for: The Effect of Monophasic Pulsed Current with Stretching Exercise on the Heel Pain and Plantar Fascia Thickness in Plantar Fasciitis: A Randomized Controlled Trial
Source: Healthcare (Basel). 2020 Mar 30;8(2):79. doi: 10.3390/healthcare8020079 (PMC7349871; doi:10.3390/healthcare8020079)
Supplement: Supplementary file 1 [file healthcare-08-00079-s001.pdf]

**Supplementary Materials**

- The patient will be instructed to cross the affected leg over the sound leg while seated, and using his/her hand, applying metatarso-phalangeal joint dorsiflexion (pulling the toes back toward the shin until the patient feels a stretch in the arch of the foot).
- Hold each stretch for a count of 10 (or 10 seconds), and repeating 10 times (DONOT OVER STRETCH).
- All patients will be asked to perform the stretching program three times per day.
- Keep a daily log of stretching exercise for 4 weeks.
- The first stretch will be done before taking the first step in the morning.

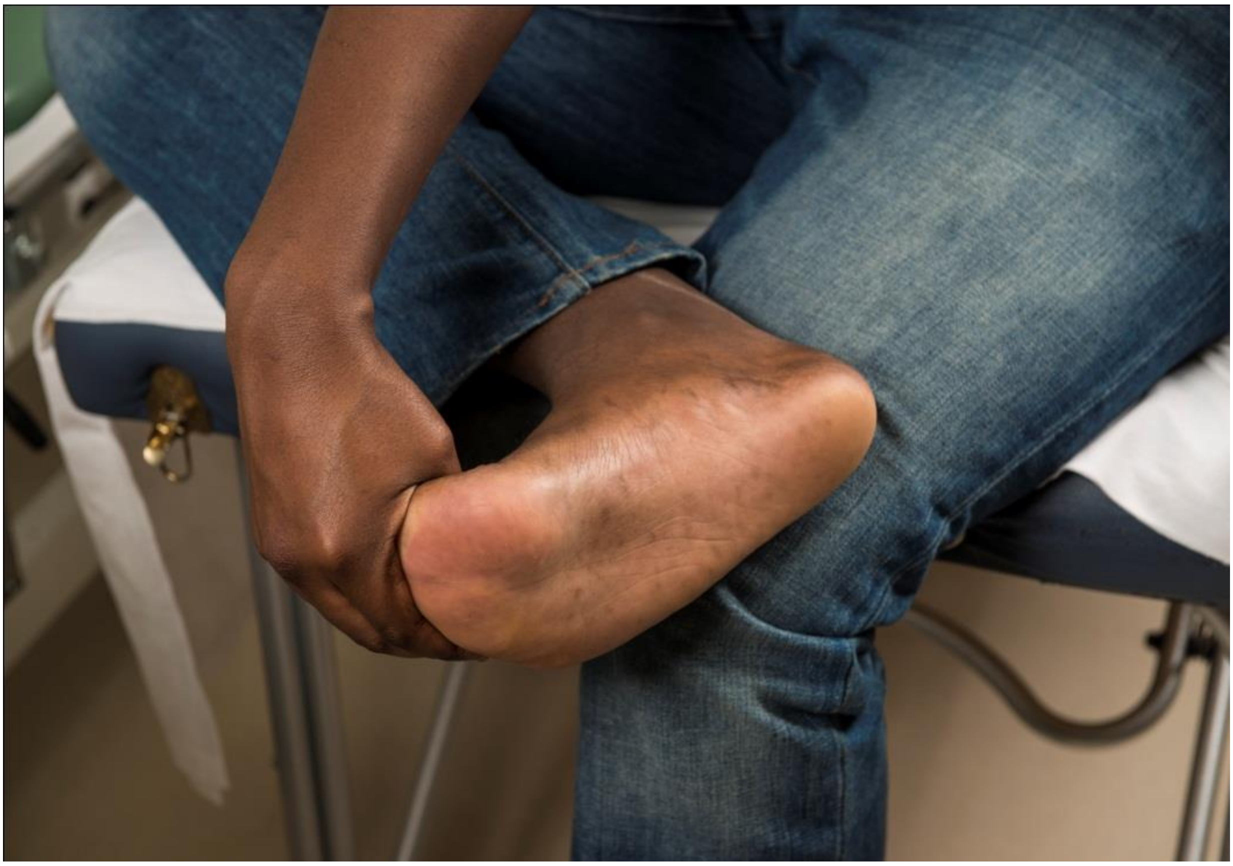

**Figure S1.** Home based plantar fascia stretching exercises.

**Table S1.** Home based plantar fascia stretching exercises log. Plantar Fascia stretching exercises log.

Name: -------------------------------------------------

| Day / Week | Monday | Tuesday | Wednesday | Thursday | Friday | Saturday | Sunday |
|---|---|---|---|---|---|---|---|
| Week 1 | | | | | | | |
| Week 2 | | | | | | | |
| Week 3 | | | | | | | |
| Week 4 | | | | | | | |
